# Pandora's Box

**DOI:** 10.1192/bji.2020.47

**Published:** 2020-11

## Are your children having screen time or green time?

The widespread availability of screen-based technology has increasingly engaged young people, to the extent that screen-based activity has significantly overtaken green time, that is, interaction with nature. It is highly likely that this may have adverse effects on children's well-being and mental health. The Covid pandemic and the associated lockdown have made matters worse.

The authors of a systematic scoping review collated evidence from databases (PubMed, PsycInfo, Scopus, Embase) and found 186 suitable quantitative studies on the subject. They aimed to assess associations among screen time, green time and psychological outcomes, which included mental health, cognitive function and academic achievement in children <5 years of age, school children (5–11 years), early adolescents (12–14 years) and later adolescents (15–18 years). They comment on the limitations of the studies due to heterogeneity and the fact that they were mostly cross-sectional, and noted other factors. The general finding, as one would expect, was that high levels of screen time were associated with unfavourable psychological outcomes. They also considered that children from lower socioeconomic backgrounds may be disproportionately affected by high screen time and low green time, and noted that future research needs to be longitudinal and able to distinguish between passive and interactive screen activities, as well as incidental and purposive exposure to nature. They did think, however, that there, is at least preliminary evidence that green time can buffer consequences of high screen time, and concluded that nature may be an under-utilised public health resource for the psychological well-being of the young, in an increasingly high-tech world.

Oswald TK, Rumbold AR, Kedzior SGE, Moore VM. Psychological impacts of “screen time” and “green time” for children and adolescents: a systematic scoping review. PLoS ONE 2020; 15(9): e0237725.3288666510.1371/journal.pone.0237725PMC7473739

## Is bilingualism good for children?

A substantial number of families around the world are of mixed ethnicity and may speak two different languages. This can be the source of a dilemma for parents wondering whether they should choose one language for their children, in the hope they may be interested in learning the other one later on, or encourage them to learn both languages from the start. Will learning two languages confuse their children or affect their ability to speak any language, or even cause them to speak in a mixture of languages in an Esperanto fashion? Well, for those of you facing such worries, a recent study will reassure you that learning two languages is not bad for your child.

It is known that in adults bilingualism does affect brain structure, with experience-dependent grey and white matter changes in those brain structures that are involved in language learning, processing and control. In a cross-sectional study comparing monolingual and bilingual youngsters, the researchers examined the developmental patterns of grey matter (thickness, volume and surface area) and white matter (fractional anisotropy and mean diffusivity) structures, across cortical and subcortical brain structures and tracts. They found that compared with monolingual youngsters, bilinguals had more grey matter (that is, less developmental loss) starting during late childhood and adolescence in the frontal and parietal regions of the brain, and higher white matter integrity (that is, a greater developmental increase) starting in mid-late adolescence, specifically in the striatal-inferior frontal fibres.

These findings indicate that the bilingual brain does indeed differ from the monolingual brain, and that this difference begins to be apparent even during development. So, whether your child is bilingual in childhood or adulthood, he or she will still have a different brain from monolinguals, and this is not a bad thing. They are essentially more ‘brainy’ as far as language function is concerned!

Pliatsikas C, Meteyard L, Veríssimo J, Vincent DeLuca, Shattuck K, Ullman MT. The effect of bilingualism on brain development from early childhood to young adulthood. Brain Struct Funct 2020; 225: 2131–52.3269121610.1007/s00429-020-02115-5PMC7473972

## Whether you are emotional or cool is all down to your brain

If you find yourself bursting in tears when watching a sad film, you are considered a person with high emotional (affective) empathy, whereas if you keep cool but remain empathetic, you are classed as high in cognitive empathy.

A meta-analysis of 40 functional magnetic resonance imaging (MRI) studies showed that affective empathy is associated with increased activity in the insula, whereas cognitive empathy is associated with activity in the mid-cingulate cortex and dorsomedial prefrontal cortex. However, it is not clear whether brain morphology actually determines the disposition to affective or cognitive empathy. In order to assess this, the researchers recruited 176 individuals, who were asked to complete a questionnaire on cognitive and affective empathy (QCAE); all participants underwent an MRI study in which high-resolution three-dimensional T1 weighted structural scans were acquired.

Those with high scores on affective empathy, as per the QCAE, had greater grey matter density in the insular cortex, and those with high cognitive empathy scores had greater grey matter density in the mid-cingulate cortex and dorsomedial prefrontal cortex. Given our brains’ capacity for neuroplasticity, can people be trained to be more empathetic? Further studies planned by the authors may answer this question.

Eres R, Decety J, Louis WR, Molenberghs P (2015) Individual differences in local gray matter density are associated with differences in affective and cognitive empathy. NeuroImage 15; 117: 305–10.10.1016/j.neuroimage.2015.05.03826008886

## Is doing good, good for you?

Being kind or charitable (prosocial) is a human quality that can help others in society at times of need; it has indeed been invaluable during the Covid pandemic and the resulting lockdown and other restrictions. But is it good for you? The evidence suggests that, yes, it is good for one's well-being and associated with better mental and physical health; however, it is not known how strong the relationship is between doing good and feeling good and what influences this.

In a recent meta-analysis, the authors examine the strength of the link between prosociality and well-being, and also how theoretical, demographic and methodological variables may moderate this link. Analysing data from 201 studies with a total of 198 213 participants, they found a modest but meaningful effect size between prosociality and well-being. Examining the data in more detail, they noted that there was a stronger link between kindness and ‘eudaimonic’ well-being (focusing on self-actualisation) compared with ‘hedonic’ well-being (feeling happy). They also observed that informal helping of others, such as random or spontaneous acts of kindness, is more likely to be associated with well-being than formal help such as organised volunteering for a charity.

Hui BPH, Ng JCK, Berzaghi E, Cunningham-Amos LA, Kogan A. Rewards of kindness? A meta-analysis of the link between prosociality and well-being. Psychol Bull 2020 Available from: 10.1037/bul0000298.32881540

## Mental health and suicide prevention – are we making progress?

Every year on 10 September, the day designated by the World Health Organization (WHO) as World Suicide Day, we are reminded that this major cause of death remains a global challenge. Close to 800 000 people die by suicide every year. Every 40°s, someone, somewhere in the world, takes his or her own life, and for each one of these suicides, there are more than 20 suicide attempts.

In 2013, the WHO launched its ambitious 7-year Mental Health Action Plan, which was adopted by the 66th World Health Assembly. This was described by the WHO Director General, Dr Margaret Chan, as a landmark achievement. Mental health, the Cinderella of medicine, was finally getting to go to the ball, and the Action Plan was received with hope and enthusiasm across the world. It made great promises, including the reduction of suicide globally by 10% by the year 2020 (Global Target 3.2 of the Mental Health Action Plan).

Have this and the other targets of the Action Plan been reached? Time will tell, when the evaluation of this programme, hopefully starting next year, is published. However, so much has happened in the past few years that may have made these ambitious targets even less likely to be achieved. Conflicts in various parts of the world, increasing number of refugees and people displaced within and outside their countries, oppression of ethnic and religious minorities, and major ecological disasters have made the task even more difficult. The Covid pandemic is the final straw.

The very existence of the WHO is threatened, particularly since the pandemic. The US, which had been the major financial contributor to the WHO, has withdrawn its support. It is a paradox that despite enormous scientific and technological progress, human behaviour seems to have taken a turn backwards, close to medieval ways of thinking in that power is valued more than social conscience and human rights. A new world order seems to be emerging, and it won't be in the interests of mental health or humanity in general.

World Health Organization. *Mental Health Action Plan 2013–2020* WHO, 2013. Available from: https://www.who.int/mental_health/publications/action_plan/en/.

## What might happen after the Covid pandemic?

It is being recognised that there may be many changes in the way we go about our personal and work lives post-Covid, but could there be more dramatic changes and events? Epidemics don't just have health implications; they have the potential to bring about major sociopolitical changes. This issue is examined by two Italian experts using a historical analysis. The main conclusions are that although during the epidemic there is relative peace, dissatisfaction is growing, and this may finally explode after the epidemic.

The Covid-19 pandemic has overshadowed major protests such as ‘Black Lives Matter’, the ‘Gilets Jaunes’, environmental activism and many others. A Freedom House annual report states that of the 20 protest movements that were active worldwide in December 2019, only two or three remain active. Yet, a lot of discontent is brewing, with ongoing serious ill-health and substantial numbers of deaths, the imposed ‘lockdowns’ and distancing restrictions with their associated psychological and social consequences and economic hardship; at the same time, an attitude of denial is shown by followers of the ‘virus conspiracy’ theory promoted by some, including powerful politicians.

The authors quote past situations of epidemics or pandemics that led to major sociopolitical unrest and argue that these can provide reliable information on similar possible effects of the current pandemic. A good example, going far back to the 14th century, is the uprising in England, France and Italy that followed the Black Death (1346–1353). The authors identified the most significant 57 epidemics over the period between the Black Death and the Spanish Flu (1919–1920) and found that in all but four cases, revolts that took place during the period of the epidemic were mainly connected to the disease. Any pre-epidemic grievances were ‘crowded out’. However, they point out that the epidemic period may be actually acting as a ‘social incubator’ for more serious disorders and, based on historical evidence, they claim that the epidemics display a ‘potential disarranging effect on civil society’. This, they note, occurs along three dimensions: (a) the policy measures tend to conflict with people's interests and facilitate attrition between society and institutions; (b) the differential effects of the epidemic on society in terms of mortality and economic status exacerbates inequality; and (c) there may be irrational narratives on the causes and the spread of the disease, which may lead to social and racial discrimination and xenophobia. These phenomena may be moderated or exacerbated by the degree of social cohesion and political stability, the duration and extent of morbidity and mortality, and how the socioeconomic costs are distributed in the society. In their attempt to check the potential of social incubation during an epidemic leading to post-epidemic unrest, the authors examined five cholera epidemics in different parts of the world. Computing the episodes of revolt in the 10 years before and the 10 years after an epidemic, they identified 39 revolts before and almost twice as many (71 revolts) after an epidemic.

Another issue they considered important is the post-epidemic repercussions of the necessary restrictions of freedom during an epidemic. These may be exploited by governments to reinforce their power, and there may be dramatic divisions along ethnic or political and economic lines, with the likelihood of repression increasing. Those in power can justify interventions to stop protest gatherings with the possibility of contagion. Protesting *per se* is less likely to occur owing to personal fear of contagion and greater tolerance of personal privacy with the need for tracking and tracing. All of these weaken any protests and opposition, and serve to consolidate power and the status quo.

Censolo R, Morelli M. COVID-19 and the potential consequences for social instability. Peace Econ Peace Sci Public Policy 2020 Available from: 10.1515/peps-2020-0045.

